# Human prostate organoid generation and the identification of prostate development drivers using inductive rodent tissues

**DOI:** 10.1242/dev.201328

**Published:** 2023-07-12

**Authors:** Parmveer Singh, Nadia A. Lanman, Hannah L. R. Kendall, Laura Wilson, Ryan Long, Omar E. Franco, Adriana Buskin, Colin G. Miles, Simon W. Hayward, Rakesh Heer, Craig N. Robson

**Affiliations:** ^1^ Translational and Clinical Research Institute, Newcastle University Centre for Cancer, Newcastle University, Newcastle upon Tyne, NE2 4AD, UK; ^2^ Department of Comparative Pathobiology, Purdue University, West Lafayette, IN 47907, USA; ^3^ Purdue University Center for Cancer Research, Purdue University, West Lafayette, IN 47907, USA; ^4^ Department of Surgery, NorthShore University HealthSystem, Evanston, IL 60201, USA; ^5^ University of Chicago Pritzker School of Medicine, Chicago, IL 60637, USA; ^6^ Translational and Clinical Research Institute, Newcastle University, Central Parkway, Newcastle upon Tyne, NE1 3BZ, UK; ^7^ Department of Urology, Freeman Hospital, The Newcastle upon Tyne Hospitals NHS Foundation Trust, Newcastle upon Tyne, NE7 7DN, UK

**Keywords:** Prostate development, Organoids, Urogenital sinus, Seminal vesicle, Human, Rat

## Abstract

The reactivation of developmental genes and pathways during adulthood may contribute to pathogenesis of diseases such as prostate cancer. Analysis of the mechanistic links between development and disease could be exploited to identify signalling pathways leading to disease in the prostate. However, the mechanisms underpinning prostate development require further characterisation to interrogate fully the link between development and disease. Previously, our group developed methods to produce prostate organoids using induced pluripotent stem cells (iPSCs). Here, we show that human iPSCs can be differentiated into prostate organoids using neonatal rat seminal vesicle mesenchyme *in vitro*. The organoids can be used to study prostate development or modified to study prostate cancer. We also elucidated molecular drivers of prostate induction through RNA-sequencing analyses of the rat urogenital sinus and neonatal seminal vesicles. We identified candidate drivers of prostate development evident in the inductive mesenchyme and epithelium involved with prostate specification. Our top candidates included *Spx*, *Trib3*, *Snai1*, *Snai2*, *Nrg2* and *Lrp4*. This work lays the foundations for further interrogation of the reactivation of developmental genes in adulthood, leading to prostate disease.

## Teaser

**Summary:** Analysis of prostate organoids generated by co-culture of rat seminal vesicle mesenchyme with human iPSCs *in vitro* and RNA sequencing of rat tissue known to induce prostate development identifies candidate drivers of prostate development.

## INTRODUCTION

The intricacies of prostate development have yet to be completely elucidated; however, drivers of the process are continually uncovered using innovative methods. Further interrogation of the developing prostate will not only benefit our knowledge of development, but may also provide some insights into prostate diseases, such as prostate cancer and benign prostatic hyperplasia (BPH), given that similarities have been observed between the molecular and histological profiles of developing and diseased prostates (Buskin et al., 2021). It is of particular interest to increase understanding of developmental pathways that may be reactivated in the growth-quiescent mature prostate, leading to disease.

One of the earliest concepts linking prostate development and disease was proposed by John McNeal (McNeal, 1978), who hypothesised that signalling pathways that are primarily active during prostate development, specifically in the stroma, become reactivated during adulthood leading to BPH. This idea of ‘embryonic reawakening’ may also apply to some forms of prostate cancer that share similarities in molecular profiles (Schaeffer et al., 2008; Pritchard et al., 2009; Buskin et al., 2021). The hypothesis suggests that the BPH stroma is inductive and that the adult epithelium is responsive to the inductive cues (Cunha, 2008). Recombination experiments using stromal cells from either BPH or prostate cancer specimens have supported the first of these two notions (Barclay et al., 2005). In contrast to normal prostate stromal cells, when stromal cells from prostate cancer-associated fibroblasts (CAFs) are recombined with immortalised prostate epithelial cells followed by renal capsule grafting, they produce highly disorganised and overgrown grafts that invade host renal tissue (Olumi et al., 1999; Hayward et al., 2001; Barclay et al., 2005). Further recombination experiments support the second notion underlying the hypothesis of embryonic reawakening – that adult epithelium is responsive to inductive cues. Human BPH epithelium xenografts undergo extensive growth and branching morphogenesis when recombined with rat urogenital sinus mesenchyme (UGM) (Hayward et al., 1998).

Subsequent studies explored the similarities between the molecular profiles of the developing and diseased prostate. For example, comparisons of microarray profiles revealed a subset of genes that were similarly expressed in both human pubertal prostate and BPH tissue, but not in the normal adult prostate (Dhanasekaran et al., 2005). Profiling of human foetal prostate stroma, normal adult prostate stroma, and CAFs also identified a subset of genes that were similarly co-expressed in foetal stroma and CAFs (Orr et al., 2012; Nash et al., 2018). More in-depth studies have utilised the developing rodent prostate at multiple stages in order to generate developmental signatures, which were also shown to be enriched in various grades of prostate cancer (Schaeffer et al., 2008; Pritchard et al., 2009). In some instances, signalling pathways, such as E-cadherin (cadherin 1) and IL1, have been functionally linked with both prostate development and disease in loss- or gain-of-function studies (Jerde and Bushman, 2009; Keil et al., 2014). Thus, developmental signalling pathways warrant further investigations as they may lead to the identification of therapeutic targets for prostate cancer and/or BPH.

Because of the limited availability of human foetal prostate tissue, especially at early stages of development, many transcriptomic profiling studies of prostate development have used the developing rodent prostate as a model system. Although developmental cues generally appear to be conserved between species, at least at earlier stages, it is well established that the adult human and rodent prostates differ in overall anatomy (McNeal, 1981), epithelial cell content (El-Alfy et al., 2000) and stromal layer thickness (Hayward et al., 1998). The pioneering work led by Gerald Cunha established that cues from the rodent UGM lead to prostate differentiation of the urogenital sinus epithelium (UGE), as well as other endodermal-derived urogenital epithelium tissues (Cunha, 1972; Donjacour and Cunha, 1993). Neonatal seminal vesicle mesenchyme (SVM) can also generate prostate tissue when recombined with endodermal-derived urogenital epithelium at a similar frequency, indicating that the UGM and SVM express a similar array of developmental cues (Donjacour and Cunha, 1995). However, when the UGM or SVM is recombined with mesodermally derived seminal vesicle epithelium (SVE), seminal vesicle differentiation occurs, indicating that the epithelium responds selectively to cues from inductive mesenchyme. Crucially, recombination studies have shown that rodent UGM or SVM can induce prostate differentiation of human epithelium, and human foetal UGM can generate prostate tissue from rodent endodermal epithelium, consistent with the conservation of developmental pathways for prostate specification (Cunha et al., 1983; Aboseif et al., 1999; Cunha et al., 2021). Recent studies have extended these findings and reported the generation of human prostate tissue by recombination between either rodent UGM or SVM and human embryonic stem cells (hESCs) or induced pluripotent stem cells (iPSCs) (Taylor et al., 2006; Hepburn et al., 2020). Co-culturing rodent UGM with human iPSCs *in vitro* can generate prostate organoids that can be genetically modified to study prostate cancer (Hepburn et al., 2020). To realise their full potential, human iPSC-based prostate induction approaches need both technological advancement and increased molecular understanding.

A number of studies have profiled the developing prostate in order to elucidate the regulators of organ development (Abbott et al., 2003; Zhang et al., 2006; Schaeffer et al., 2008; Pritchard et al., 2009). Although these studies provided some initial insights into gene expression changes during prostate development, there was an apparent lack of sensitivity resulting in a failure to highlight many previously identified prostate developmental genes. This was likely due to the limitations of the profiling methods utilised as well as each prostate being assessed as a whole rather than distinct tissue types (e.g. UGM and UGE), resulting in the dilution of signals of genes expressed at low levels in specific tissue subsets (de Bruin et al., 2005).

In the current study, two approaches were taken to expand our knowledge of prostate disease and development based on the inductive UGM and SVM. First, we successfully generated prostate organoids from iPSCs by co-culture with SVM, providing an alternative to UGM for generating prostate organoids, which can be difficult to isolate for those with little microdissection experience. These organoids can be used to study prostate differentiation or modified to study prostate cancer. Second, the ability of both UGM and SVM to induce prostate differentiation provided an opportunity to identify key regulators of prostate development by comparing upregulated genes in both tissue subsets. Additionally, the difference in response of the UGE and SVE to cues from the mesenchymal tissues indicates a difference in pathway activation in the epithelia. RNA sequencing (RNA-seq) was employed for the first time to profile mesenchymal and epithelial subsets of the rat UGS immediately prior to budding as well as neonatal seminal vesicles. Because the UGM and SVM can both drive prostate differentiation, candidate genes and pathways upregulated in these tissues were identified that may be associated with prostate specification. Additionally, given that the UGE and SVE respond differently to cues from the mesenchyme, we identified genes that may be necessary for prostate specification over seminal vesicle when responding to mesenchymal signals.

## RESULTS

### Human iPSC-derived prostate organoids can be generated from SVM

Previous work demonstrated the ability of SVM to generate prostate tissue when recombined with hESCs and renal capsule grafted into rodents (Taylor et al., 2006). A major limitation of this method was the need for xenografting the SVM–hESCs cell recombinants for *in vivo* growth and differentiation. Prostate tissue organoids can be generated wholly *in vitro* when cultured in 3D conditions with rat UGM (Hepburn et al., 2020); however, it remains unclear whether rat SVM can similarly induce iPSCs *in vitro*.

**Fig. 1.**
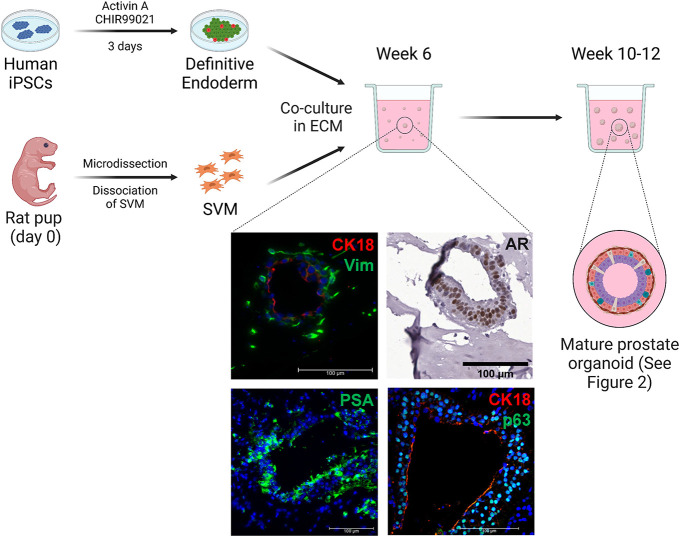
**The process of generating prostate organoids from SVM-iPSC co-cultures.** Human iPSCs were differentiated into definitive endoderm using activin A and CHIR99021. Seminal vesicle mesenchyme (SVM) was isolated from rat pups. Definitive endoderm and SVM cells were co-cultured in Corning Matrigel (ECM). By 6 weeks, epithelial organoids were visible and surround by vimentin (Vim)-positive mesenchymal cells. Androgen receptor (AR) was expressed in almost all epithelial cells. PSA was detected around luminal cells. A high ratio of p63-expressing cells to CK18-expressing cells was observed. By week 12, the prostate organoids had matured and the cellular composition resembled an adult human prostate gland (see Fig. 2). Created with BioRender.com.

**Fig. 2.**
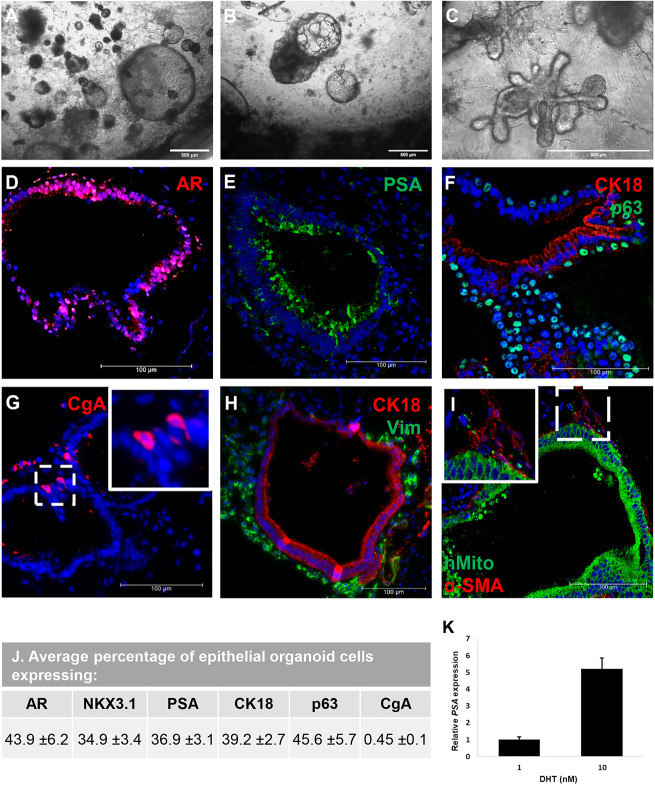
**Prostate organoids generated from iPSCs using SVM.** (A-I) Definitive endoderm cells derived from human-iPSCs were co-cultured with rat pup SVM in Corning Matrigel for a period of 12 weeks. (A) Spherical structures formed with lumen. Some of these structures developed buds (B) and elongated/branched (C). The organoids contained AR- and PSA-expressing luminal cells (D,E), p63-positive basal cells (F), CK18-positive epithelial cells (F,H), rare chromogranin A (CgA)-positive neuroendocrine cells (G), vimentin (Vim)-positive stromal cells (H), and all cells expressed a human mitochondrial marker (hMito; I) and smooth muscle cells (α-SMA; I). Nuclei were stained with DAPI (blue). (J) The percentage of organoid epithelial cells expressing each marker was estimated by counting cells of 150 distinct organoid sections from three cultures. (K) PSA expression was measured after increased DHT exposure for 7 days to assess organoid response. Error bars represent s.e.m.

**Fig. 3.**
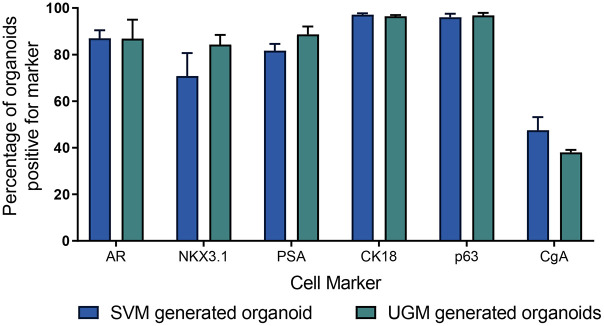
**Mature SVM-iPSC prostate organoids express cell markers similarly to UGM-iPSC prostate organoids.** The number of organoids expressing AR, NKX3­1, PSA, CK18, p63 or CgA were counted and compared with the total number of organoids in order to calculate the percentage of organoids expressing each prostate marker. An organoid was considered to be positive for a marker if it showed a similar expression pattern of the marker compared with a human prostate gland. Organoids were generated using either SVM-iPSC (*N*=3 cultures, *n*=201 organoids counted in total) or UGM-iPSC (*N*=3 cultures, *n*=178 organoids counted in total) co-cultures. Error bars represent s.e.m.

Co-culturing SVM and definitive endoderm generated from iPSCs in Matrigel and prostate organoid medium containing dihydrotestosterone (DHT) resulted in the formation of spherical structures (Figs 1,2A). These structures would spontaneously bud, elongate and branch, although budding and branching were rare events with approximately 8% of mature organoids showing buds and 11% elongation or branches at a given time (Fig. 2B,C, Table 1). By week six of co-culture, basal and luminal layers could be distinguished with a higher proportion of basal cells compared with luminal; androgen receptor (AR) was expressed in most epithelial cells, and prostate specific antigen (PSA; also known as KLK3) was observed around luminal cells (Fig. 1). Cell types such as neuroendocrine and α-smooth muscle actin (α-SMA)-expressing cells were not detected in week six organoids. After 10-12 weeks of culture, the organoids were composed of AR- and PSA-expressing luminal cells (Fig. 2D,E) surrounded by p63 (TP63)-positive basal cells (Fig. 2F) and rare CgA (CHGA)-positive neuroendocrine cells (Fig. 2G). The ratio of luminal to basal cells was estimated to be equal in mature organoids (Fig. 2J). Surrounding the glandular epithelial organoids were layers of stromal cells (Fig. 2H), some of which differentiated to express α-SMA (Fig. 2I). The organoids and attached stroma were confirmed to be derived from the human iPSCs by positive staining for the human-specific mitochondrial marker [113-1] (Fig. 2I). Assessment of the number of organoids and cells expressing the markers above indicated that the differentiation efficiency was similar to organoids generated using UGM (Figs 2J, 3) (Hepburn et al., 2020). An organoid was considered to be positive for a marker if it showed a similar expression pattern of the marker compared with a human prostate gland. Additionally, the frequency of SVM-generated organoids with lumen was similar to that of UGM-generated organoids (Table 1). However, SVM-generated organoids on average had higher frequencies of budding and branching organoids. Lastly, mature organoids were stimulated with varying concentrations of DHT (1 nM and 10 nM) to show that they were functionally responsive. Compared with 1 nM, 10 nM DHT resulted in approximately five times more PSA expression (Fig. 2K) for a period of 7 days.

**Table 1.**
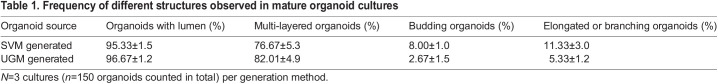
Frequency of different structures observed in mature organoid cultures

### RNA-seq analysis of inductive tissues detects drivers of prostate development

**Fig. 4.**
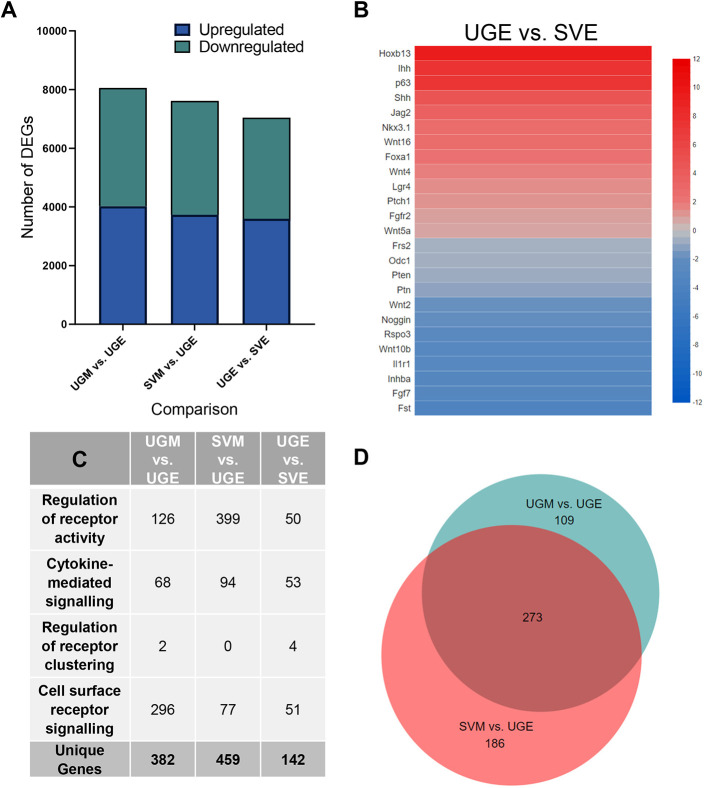
**Identification of prostate developmental genes.** (A) Statistically significant (FDR≤0.05) differentially expressed genes (DEGs) were identified between mesenchymal and epithelial subsets (*N*=3 for each tissue subset). (B) Genes that have been functionally associated with prostate development were identified in DEG datasets to generate heatmaps to illustrate prostate developmental genes that are likely necessary for prostate specification over seminal vesicle specification (data represent Log2 fold changes). (C) Upregulated DEGs associated with cell signalling based on GO annotations were determined for the two DEG analyses. (D) The resulting gene lists were compared to identify common genes involved in cell–cell signalling. Created with the BioVenn application (https://biovenn.nl/; Hulsen et al., 2008).

To identify the molecular pathways involved in prostate induction, RNA-seq was performed on isolated male UGM and UGE of embryonic day (E) 18 rats as well as the SVM and SVE of day 0 rat pups. For each sample, a minimum of 50 million reads was achieved (*N*=3 for each tissue type). At least 95% of the reads passed quality control checks, and of those reads passing quality control over 92% were mapped to the reference genome (Table S1). Reads that were uniquely mapped to annotated genes were used to generate a count matrix for each sample (Table S2). Expression profiles were then compared with each other to generate lists of differentially expressed genes (DEGs). The aim of this study was to uncover candidate genes involved in prostate development with a focus on paracrine factors leading to prostate induction and budding of the epithelium. Because the UGM and SVM can both induce prostate differentiation, we hypothesised that these two tissues express a similar set of genes involved in prostate induction. Accordingly, we generated lists of statistically significant (false discovery rate, FDR≤0.05) DEGs between mesenchymal and epithelial subsets (Fig. 4A; Tables S3, S4, S5). Additionally, UGE-enriched transcripts were assessed relative to the SVE. The UGE produces prostate when recombined with UGM or SVM, and the SVE forms seminal vesicle when exposed to UGM or SVM, so these tissue types are either selective to the cues to which they respond or respond differently to the same cues. Thus, we hypothesised that differentially enriched receptors between these two epithelia may elucidate the pathways involved in prostate specification.

**Fig. 5.**
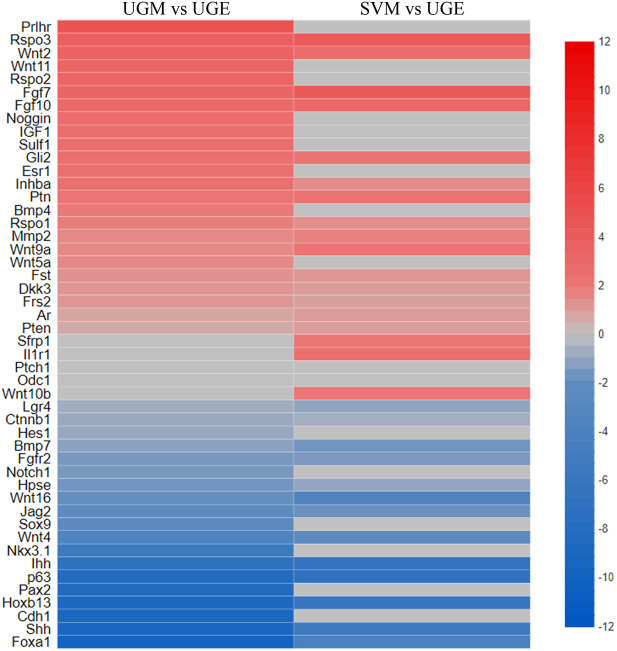
**RNA-seq profiling of the UGM, UGE and SVM detects known prostate developmental genes in the expected tissue subsets.** Statistically significant (FDR≤0.05) differentially expressed genes (DEGs) were identified between mesenchymal and epithelial subsets (*N*=3 for each tissue subset). Genes that have been functionally associated with prostate development were identified in DEG datasets to generate heatmaps to illustrate their relative expression between tissue subsets (data represent Log2 fold changes).

**Fig. 6.**
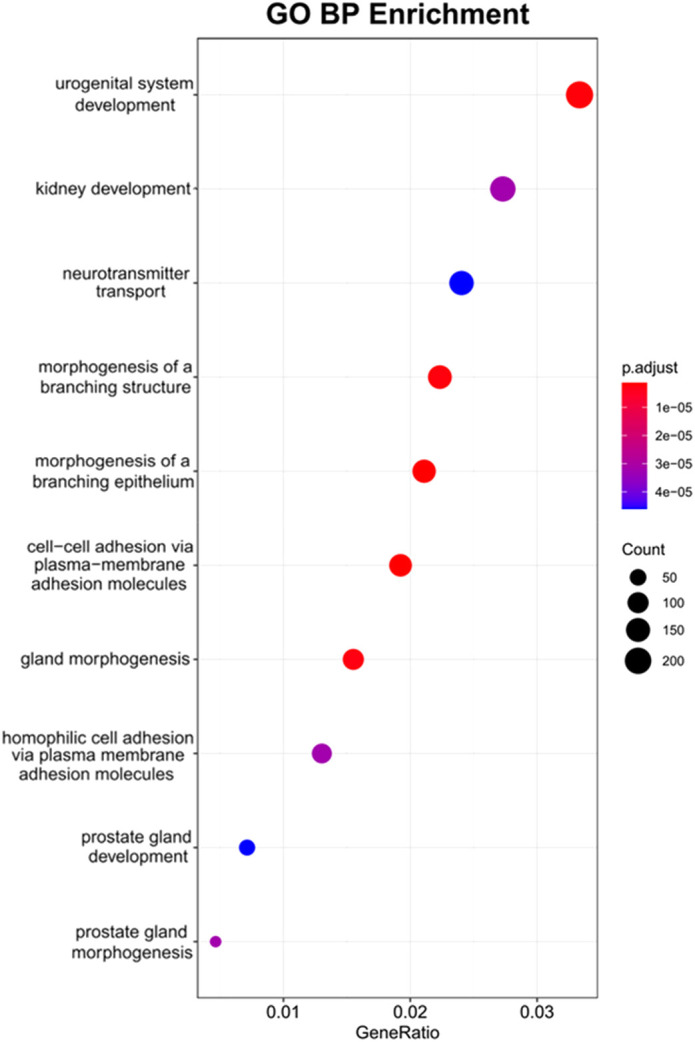
**Top GO biological processes for UGM-UGE comparison.** DEGs upregulated in UGM showed enrichment for GO terms related to prostate development.

The DEG lists revealed numerous genes that have previously been associated with prostate development and were enriched in either the UGM or UGE, as expected (Fig. 5), confirming that the experimental designs and sequencing depth were sensitive enough to detect important developmental genes, including transcription factors. For example, *Ar*, *Bmp4*, *Fgf7*, *Fgf10*, noggin and proteins downstream of Hedgehog signalling were all enriched in the UGM, whereas *Fgfr2*, *Foxa1*, *Hoxb13*, *Sox9*, *Shh*, *Ihh* and *Nkx3-1* were enriched in the UGE. Several of the UGM-enriched transcripts were also enriched in the SVM, as would be expected from factors involved in prostate induction. Additionally, some of the top biological processes from a Gene Ontology (GO) analysis of the UGM-UGE dataset were related to prostate development (Fig. 6). Furthermore, a number of prostate developmental transcripts were found enriched in the UGE relative to the SVE, likely signifying their importance to prostate specification over seminal vesicle development (Fig. 4B).

### Identification of candidate prostate developmental genes

To narrow down the list of candidate developmental genes, we focused on upregulated genes (fold change, FC≥1.5) associated with cell signalling as we were interested in paracrine factors and receptors that may be expressed during prostate specification. Therefore, we investigated genes that fell into the following GO term categories: ‘regulation of receptor activity’, ‘cytokine-mediated signalling pathway’, ‘regulation of receptor clustering’ and ‘cell surface receptor signalling pathways involved in cell signalling’. Fig. 4C lists the number of transcripts that fell into each of these categories that were enriched in either the UGM or SVM relative to the UGE, and in the UGE relative to the SVE (Tables S6, S7, S8). The number of unique genes across the categories are also listed for each DEG comparison (Fig. 4C). To narrow down the list of candidate genes, we considered the 273 cell signalling genes upregulated in both the UGM and SVM because both tissues express a set of genes responsible for prostate differentiation (Fig. 4D; Table S9). This resulting list contained some of the known prostate developmental mediators, including FGF and Wnt family genes.

Additional analyses were conducted using Ingenuity Pathway Analysis software (IPA, QIAGEN Inc., https://digitalinsights.qiagen.com/IPA) to identify upstream regulators that may be associated with prostate development. The analysis included DEGs with a Log2FC of greater than 1.5 or −1.5. Of the resulting DEGs, over 92% were mapped to the IPA knowledgebase for each comparison. The top 50 predicted activated upstream regulators enriched in UGM relative to the UGE are listed in Table S10. For the UGE-SVE comparison, the upstream analysis was focused on enriched receptors and transcription regulators (Table S11). Lastly, a receptor–ligand analysis was conducted between the UGM and UGE datasets to identify any paracrine signalling that might be occurring between the two tissues (Table S12).

Using the lists of genes generated from these analyses and assessing the known roles and functions of the genes, we were able to identify several candidate genes of interest that may be involved in prostate development. Mesenchymal upregulated candidate genes included *Spx*, *Trib3*, *Snai1*, *Snai2*, *Nrg2*, *Rln1*, *Gdf9*, *Gdf5*, *Hgf*, *Dact3*, *Sfrp4*, *Etv2*, *Dkkl1* and *Shisa7*. Epithelial upregulated genes included *Lrp4*, *Pax9*, *Yap1*, *Dlx5*, *Musk*, *Palm3*, *Lefty1*.

Analysis of cell–cell signalling genes as well as the receptor–ligand analysis identified neuregulin 2 (*Nrg2*) as a ligand of interest. *Nrg2* was upregulated in the UGM whereas genes encoding receptors of NRG2, *Erbb2* and *Erbb3*, were enriched in the UGE. *Nrg2* has been associated with growth and differentiation of epithelial cells (Ring et al., 1999). Another member of the neuregulin family with some similarity, *Nrg1*, was also upregulated in the UGM and the SVM. Recently, NRG2 was shown to increase lumen size and the number of polarised luminal cells in adult human and mouse primary tissue organoids (Karthaus et al., 2020).

**Fig. 7.**
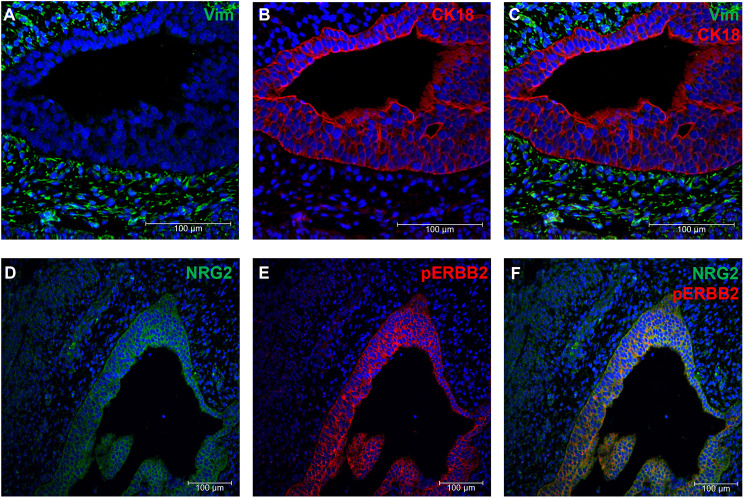
**Validation of NRG2 expression and detection of the downstream receptor ERBB2 in UGS.** (A-F) Rat E18 UGS was sectioned and stained for the mesenchymal marker vimentin (Vim) (A,C) and the epithelial marker CK18 (B,C) to show the distinct subsets. NRG2 expression was observed in both the mesenchyme and the epithelium (D,F), whereas phosphorylated ERBB2 (pERBB2) (E,F) was primarily observed in the epithelium. Nuclei were stained with DAPI (blue).

We further investigated the expression of NRG2 and activation of its downstream partners, ERBB2 and AKT, by immunohistochemical staining of the UGS and developing prostate organoids. Rat E18 UGS was first stained using antibodies against vimentin and CK18 (KRT18) to identify the distinct mesenchyme and epithelium subsets, respectively (Fig. 7A-C). NRG2 was detected in the mesenchyme, as anticipated, but also in the epithelium, indicating that it may be interacting with receptors of the UGE (Fig. 7D). Phosphorylated ERBB2 was observed in the UGE, indicating activation of the pathway, possibly through NRG2 signalling (Fig. 7E,F).

**Fig. 8.**
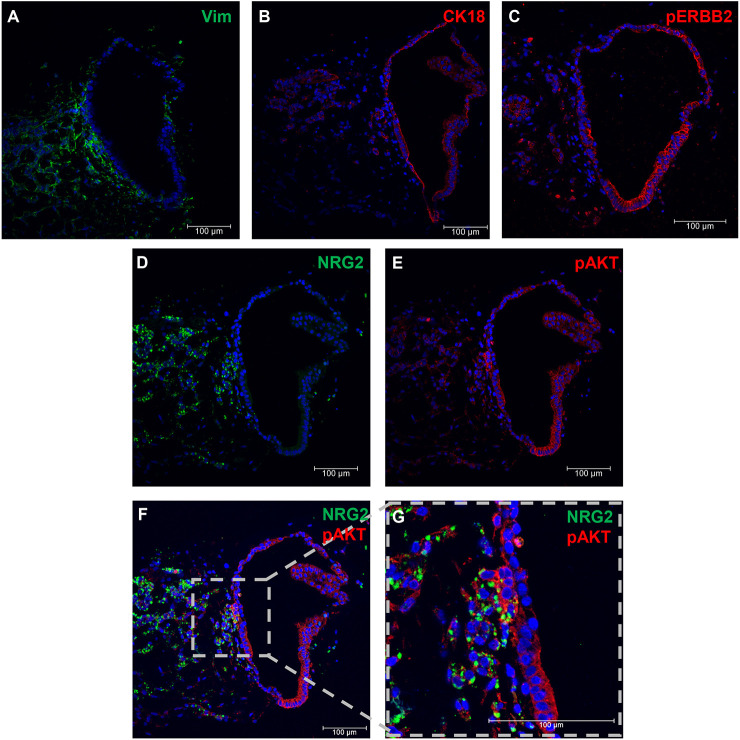
**Validation of NRG2 expression and detection of downstream proteins in developing prostate organoids.** (A-G) Week 6 iPSC-SVM-derived prostate organoids were sectioned and stained for the mesenchymal marker vimentin (Vim) (A) and the epithelial marker CK18 (B) to show the distinct subsets. NRG2 expression (D,F,G) was observed primarily in the mesenchymal subset whereas phosphorylated ERBB2 (pERBB2) (C) and AKT (pAKT) (E-G) were primarily detected in the UGE. Nuclei were stained with DAPI (blue).

Week six developing prostate organoids generated from SVM-iPSCs were also analysed by immunohistochemical staining. Vimentin and CK18 staining showed the epithelial organoid was surrounded by mesenchymal cells (Fig. 8A,B), and, similarly to the UGS, phosphorylated ERBB2 was present in the epithelial cells (Fig. 8C). NRG2 was primarily detected in the mesenchymal component and was not evident in the epithelial cells, similar to the UGS (Fig. 8D). However, we did observe NRG2-expressing mesenchymal cells closely associated with epithelial cells. Interestingly, in those epithelial cells, there was a higher level of phosphorylated AKT, as indicated by fluorescence staining (Fig. 8F,G).

## DISCUSSION

The parallels between prostate development and disease warrant further investigation into the key genes and pathways driving the process. Human iPSC-derived prostate organoids could provide a route to studying different stages of human prostate differentiation when foetal material is unavailable. The ability to generate human-like prostate organoids completely *in vitro* using SVM, as shown here, also provides an opportunity to study development in a high-throughput manner. We showed that organoids could be generated from SVM at similar efficiencies to our previously published protocol using UGM (Hepburn et al., 2020). Interestingly, prostate organoids generated using SVM compared with UGM had a higher frequency of budding and branching organoids, likely attributable to differences in the paracrine signalling factors between the UGM and SVM, suggesting that SVM-derived organoids may be a superior system in which to study branching morphogenesis. In the budding rat prostate and seminal vesicle, a key difference that has been observed is that prostate buds began as solid chords that later canalize, whereas seminal vesicle buds contain lumens from the start (Hayward et al., 1996). Branching morphogenesis is more intricate in the prostate in terms of the number of branch points (Hayward et al., 1996; Thomson and Marker, 2006). Thus, the differences in budding and branching frequencies in our organoids are likely attributable to differences in the paracrine signalling factors between the UGM and SVM during development.

The majority of organoids possessed multiple epithelial cell layers, although the exact number of layers varied between individual organoids and this variation was also observed in UGM-iPSC organoids and has previously been reported in primary cell-derived organoids, indicating that this may be a feature typical to *in vitro* models of this type (Karthaus et al., 2014; Hepburn et al., 2020).

One issue that may arise when using UGM or SVM to generate prostate tissue from iPSCs is the unintentional formation of other tissue types, such as seminal vesicle, as a result of the numerous developmental signals being released from these tissues. To avoid inadvertently generating other organoid types in our co-culture system, it may be beneficial to prime iPSCs so that they are directed to only produce prostate organoids. Upregulation of *NKX3-1* and/or *FOXA1* in the iPSCs during differentiation may reduce unwanted tissue/organ types. When either of these genes is knocked out in the prostate, a seminal vesicle phenotype is predominant (DeGraff et al., 2014; Dutta et al., 2016). Additionally, it has been shown that upregulating *Nkx3-1* in mature adult mouse SVE led to prostate development when recombined with UGM, whereas UGM and wild-type SVE recombinant resulted in seminal vesicle development (Dutta et al., 2016). Talos et al. found that upregulating *NKX3-1* and *FOXA1* in epithelial cells generated from iPSCs resulted in significantly more prostate differentiation when recombined with UGM and renal grafted compared with controls in which *NKX3-1* and *FOXA1* were not upregulated (Talos et al., 2017). Consequently, methods incorporating the manipulation of transcription factors in this way with co-culture induction may improve the derivation of prostate organoid cultures. Indeed, the candidate genes highlighted in this study may also prove to be promising targets for manipulation in iPSCs to determine whether they improve prostate differentiation when combined with SVM.

A major objective of the research presented here was to identify key drivers of prostate development. Although previous expression profiling analyses of the developing prostate have provided a wealth of data on potential candidates, most were unable to detect many of the known prostate developmental genes. This was largely due to limitations in genetic profiling technologies at the time leading to poor sensitivity and many studies opted to profile mesenchymal and epithelium tissues together, which may lead to masking of genes upregulated in one tissue but not the other. Therefore, we performed next generation sequencing on the transcriptome of separated mesenchymal and epithelial tissues of the UGS and on the SVM in order to study the molecular profiles of these tissues in more depth. In doing so, we were able to detect expression changes of almost all genes that have been previously associated with prostate development in specific tissue subsets. For example, *Fgf10* and *Fgf7* were upregulated in the UGM and the associated receptor was upregulated in the UGE, whereas *Shh* and *Ihh* were enriched in the UGE and the downstream hedgehog factors *Gli1* and *Gli2* were enriched in the UGM.

Given that the UGM and SVM can both induce prostate differentiation, we hypothesised that these two tissues should express a similar set of genes involved in prostate induction. Thus, comparing upregulated genes between these two tissue subsets assisted in the identification of candidates. Our top mesenchymal upregulated candidate genes included *Nrg2*, *Spx*, *Trib3*, *Snai1* and *Snai2*. NRG2 protein was validated in rodent UGS and found in both the UGM and UGE, with its downstream receptor, ERBB2, activated in the UGE. Additionally, NRG2 expression was detected in the stromal cells surrounding differentiating SVM-iPSC organoids with the downstream protein AKT also activated in the epithelium, providing support for the involvement of NRG2 in prostate development.

We also examined upregulated genes in epithelial subsets. Based on early work by Donjacour and Cunha (Cunha et al., 1991; Donjacour and Cunha, 1995), we hypothesised that these two epithelia may be differently primed to respond selectively to inductive cues from the mesenchyme. This comparison allowed us to compile a list of genes that were previously associated with prostate development and were significantly upregulated in the UGE relative to the SVE, which included *Fgfr2*, *Foxa1*, *Foxa2*, *Foxa3*, *Hoxb13*, *Ihh*, *Lgr4*, *Nkx3*-*1*, *Shh* and *p63*. We propose that expression of some or all of these genes in the epithelium is key in order for prostate rather than seminal vesicle development to occur. The importance of *Nkx3-1* and *Foxa1* has already been reported with deficiencies of either of these genes in the prostate leading to a seminal vesicle phenotype (DeGraff et al., 2014; Dutta et al., 2016). In addition to these known developmental genes, we were able to compile a list of candidate genes and upstream regulators enriched in the UGE, which included *Lrp4*, *Dlx2*, *Foxd1*, *Foxd3*, *Foxp1* and *Serpina1*.

Thus, from our profiling analysis, we were able to confirm the expression profiles of known prostate developmental genes and also uncover novel candidates. To solidify these candidates as prostate developmental genes, they must be further assessed for their functional association with prostate development, which was a limitation of the current study. The candidates could be tested through alteration of their expression in the developing rodent prostate or in the SVM-iPSC organoid model presented in this study. The prostate organoid model could also be utilised to improve our understanding of human prostate development. The rarity of human foetal prostate tissue, especially at earlier stages, has led to a large knowledge gap in relation to human prostate development. Prostate organoids derived from iPSCs could be useful for filling in these gaps by conducting profiling studies at various stages of differentiation. In some cases, it has been shown that ESC- or iPSC-derived organoids of other tissue types can be utilised to study development (Kanton et al., 2019; Tran et al., 2019).

The developmental profiling data generated in this study could also be used to improve our understanding of genes and pathways that become reactivated in adulthood, leading to prostate cancer or BPH. Although there is a discernible association between prostate development and disease, it is not clear how embryonic pathways are reawakened in adulthood. Similar to these previous studies, it would be beneficial to interrogate further the more in-depth datasets generated in our research to investigate links to prostate cancer, which may lead to the discovery of potential biomarkers and a better understanding of the mechanisms driving prostate cancer.

## MATERIALS AND METHODS

### Animal work

All animal experiments were performed in accordance with the Animal Welfare Ethical Review Board at Newcastle University or the Institutional Animal Care and Use Committee at Northshore University HealthSystem Research Institute. Embryonic male UGS was microdissected from E18 male Sprague-Dawley *Rattus norvegicus* (Harlan Laboratories Inc.) and neonatal day 0 seminal vesicles were isolated from male Wistar Han IGS *Rattus norvegicus* (Charles River Laboratories) as described previously (Lawrence et al., 2013; Hepburn et al., 2020).

### iPSC culturing and definitive endoderm generation

Prostate-derived iPSC clones were previously generated and characterised in our group (Hepburn et al., 2020). Cells were cultured on Corning Matrigel hESC-qualified Matrix in complete mTeSR1 medium (STEMCELL Technologies). To generate definitive endoderm, iPSCs were dissociated into single cells using Gentle Cell Dissociation Reagent (STEMCELL Technologies) following the manufacturer's protocol. Cells were counted, and 2×10^6^ cells were plated in Matrigel-coated wells of a 6-well plate in mTeSR1 containing 10 µM RHO/ROCK inhibitor Y-27632 (STEMCELL Technologies). After 24 h in RPMI-1640 medium (Sigma-Aldrich) containing 100 ng/ml of human recombinant activin A (AA; STEMCELL Technologies) and 6.45 µM CHIR99021 (STEMCELL Technologies) was added. At 24 h, RPMI-1640 medium containing 100 ng/ml AA and 0.2% foetal bovine serum was applied. After another 24 h, the medium was changed to RPMI-1640 containing 100 ng/ml AA and 2% foetal bovine serum. Definitive endodermal cells were ready for use the next day.

### iPSC-SVM prostate organoid co-culture

Rat SVM tissue was dissociated for 1 h at 37°C in 1900 U/ml of collagenase type I (Thermo Fisher Scientific) in RPMI-1640 medium on an orbital shaker. The dissociated tissue was filtered through a 70 µm cell strainer to obtain a single cell suspension of SVM. Definitive endodermal cells (from human iPSC cultures) were dissociated into single cells using Gentle Cell Dissociation Reagent and 50,000 SVM cells were combined with 10,000 definitive endodermal cells in 50% Growth Factor Reduced (GFR)-Matrigel (Corning) diluted in DMEM/F12 medium (Sigma-Aldrich). Droplets of 50 µl of the cell suspension were plated in wells of a 96-well plate coated with 50 µl of GFR-Matrigel. The plate was then placed in a 37°C incubator upside down and allowed to set for 30 min. Once set, 150 µl of DMEM/F12 containing 2% insulin-transferrin-selenium (ITS), 10 nM DHT and 10 µM Y-27632 was added on top. Medium was changed every other day. One week after plating cells, the medium was changed to SVM-conditioned medium. SVM-conditioned medium was prepared by culturing freshly isolated SVM tissue in DMEM/F12 containing 2% ITS and 10 nM DHT for a period of 7 days, collecting medium every 24 h. After an additional 2 weeks, human prostate organoid medium was applied (Karthaus et al., 2014). Medium was refreshed every other day.

### Immunofluorescence and chromogenic immunohistochemistry (IHC)

Organoids and tissues were fixed in 4% paraformaldehyde for 1 h at room temperature. Samples were processed through an automatic histology processor, then embedded in paraffin wax blocks for sectioning. Tissue sections were dewaxed and rehydrated followed by antigen retrieval using a decloaking chamber containing citrate buffer (10 mM citric acid, 0.05% Tween 20, pH 6.0).

For immunofluorescence IHC, slides were incubated in a blocking solution of 4% bovine serum albumin (BSA) in PBS (BSA-PBS) for 1 h, and then primary antibodies diluted in fresh 4% BSA-PBS solution were applied overnight at 4°C. Primary antibodies were: anti-AR (Proteintech, 66747-1-Ig, 1:800), anti-PSA (Dako, A056201-2, 1:10,000), anti-CK18 (Proteintech, 10830-1-AP, 1:15,000), anti-p63 (Leica Biosystems, NCl-L-p63, 1:50), anti-vimentin (Santa Cruz Biotechnology, sc-6260, 1:200), anti-human mitochondrial marker (Abcam, ab92824, 1:200), anti-chromogranin A (Abcam, Ab15160, 1:200), anti-neuregulin-2 (Santa Cruz Biotechnology, sc-398594, 1:100), phospho-HER2/ErbB2 (Cell Signaling Technology, 2243, 1:100) and phospho-Akt (Cell Signaling Technology, 3787, 1:200). Secondary antibodies were diluted in 4% BSA-PBS and applied for 30 min at room temperature. Secondary antibodies used were goat anti-rabbit Alexa Fluor 546 (Invitrogen, A11035, 1:400) and goat anti-mouse Alexa Fluor 488 (Invitrogen, A21121, 1:400). Coverslips were mounted with a drop of Fluoroshield Mounting Medium with DAPI (Abcam, ab104139). Slides were viewed using a Leica SPE confocal fluorescence inverted microscope.

For chromogenic IHC, tissue was blocked in a 3% hydrogen peroxide solution for 10 min and then 2.5% horse serum solution for 20 min. Primary anti-AR (Proteintech, 66747-1-Ig, 1:5000) was applied overnight at 4°C followed by anti-mouse HRP IgG (Vector Laboratories, MP-7402) for 30 min at room temperature. ImmPACT DAB Peroxidase HRP Substrate (Vector Laboratories, SK-4105) was applied to slides for 5 min. The slides were then counterstained with Gills II Haematoxylin and blued with Scott's tap-water. The slides were dehydrated in a series of ethanol washes ranging from 50% to 100%, followed by xylene washes. Lastly, coverslips were mounted on the slide using DPX Mountant (Sigma-Aldrich, 44581). Images of organoids and tissues were captured on a Leica Aperio system.

### RNA extraction and cDNA generation

RNA was isolated from organoids using the RNeasy Mini Kit (QIAGEN) as per the manufacturer's guidelines. The concentration and purity of RNA was assessed using the Nanodrop 2000 (Thermo Fisher Scientific). One microgram of isolated RNA was reverse transcribed using the M-MLV reverse transcription kit (Promega) as per the manufacturer's instructions.

### Quantitative PCR

In order to evaluate organoid response to DHT, quantitative PCR was performed. *PSA* and *HPRT1* expression was assessed using the PowerTrack™ SYBR Green Master Mix (Applied Biosystems) and was carried out on a QuantStudio 7 Flex Real-Time PCR machine (Applied Biosystems). Primer sequences can be found in Table S13. The 2^−ΔΔCt^ method was employed for data analysis and *HPRT1* was used for normalisation. *PSA* expression of organoids stimulated with 10 nM DHT was calculated relative to organoids stimulated with 1 nM DHT (normal culture conditions). Data represent three biological replicates.

### RNA-seq of UGS and SVs

UGS and SVs were isolated from three separate litters for each (*N*=3). After separation of mesenchymal and epithelial subsets, UGM and SVM were incubated for 4 h in DMEM/F12 medium containing 2% ITS and 10 nM DHT to promote AR signalling factors, whereas UGE and SVE were incubated in the same medium without the addition of DHT. Total RNA was isolated using the QIAGEN RNeasy Micro Kit (74004) following the manufacturer's protocol. Library construction and RNA-seq was conducted by GENEWIZ (Leipzig, Germany) using the Illumina HiSeq platform.

Rat genomic resources were downloaded from the ENSEMBL database (Rnor_6.0). The corresponding annotation file in GTF format was also downloaded from the ENSEMBL database. Sequence data quality was determined using FastQC software (version 0.11.7) (Babraham-Bioinformatics, 2010; http://www.bioinformatics.babraham.ac.uk/projects/fastqc/). Quality trimming and filtering were performed on the FASTQ files using Fastp (version 0.19.5) to remove the basecalls with a Phred33 score less than 30 and any reads shorter than 30 base pairs (Chen et al., 2018). Quality trimmed reads were mapped against the indexed reference genome using the STAR aligner (version 2.5.4b) (Dobin et al., 2013). STAR-derived mapping results and the ENSEMBL Rnor_6.0 annotation (GTF) file were used as input into the HTSeq package (version 0.7.0) to obtain the read counts for each gene feature in each replicate using HTSeq Count (Anders et al., 2015).

R version 3.5.1 was used for all analyses after count matrix generation. For DEG analysis in each comparison, genes with zero counts across all replicates (control and treatment) were discarded from the count matrix. The final combined count matrix was utilised for further differential gene expression analysis using the Bioconductor package DESeq2 (version 1.22.2) (Love et al., 2014). DESeq2 uses negative binomial distribution-based statistical models and performs specific estimate variance-mean tests. In DESeq2, an Empirical Bayes shrinkage was applied for dispersion estimation and a Wald test was subsequently used for significance testing. DESeq2 computes a *P*-value for each hypothesis test and the Benjamini–Hochberg method was used to correct *P*-values for multiple testing, controlling the FDR at 5%. A subsequent FC cut-off of 1.5 was employed to retain only those statistically significant genes that have a biologically relevant change in expression.

Pathway analysis was performed using ClusterProfiler (Yu et al., 2012). From each comparison, the genes which were differentially expressed at FDR≤0.05 and FC≥1.5 were used as an input for pathway analysis. A list of enriched GO categories was obtained from the ClusterProfiler results (FDR<0.05).

In order to identify upstream regulators, data were further analysed using IPA. For each analysis, DEGs with FDR≤0.05 and Log2FC≥1.5 or Log2FC≤−1.5 were included in the analysis. Prediction confidence was set to ‘experimentally observed’ and ‘high (predicted)’. Knowledge from all species was used in the analysis. For all other parameters, default settings were applied.

## Supplementary Material

10.1242/develop.201328_sup1 Supplementary information
