# Supplementary material for: Human prostate organoid generation and the identification of prostate development drivers using inductive rodent tissues
Source: Development. 2023 Jul 12;150(13):dev201328. doi: 10.1242/dev.201328 (PMC10357030; doi:10.1242/dev.201328)
Supplement: Supplementary information [file develop-150-201328-s1.pdf]

Table S1. RNA-sequencing read count summary

Sample	Total Reads	Reads Passing QC	Reads Passing QC (%)	Total Reads Mapped	Reads Mapped (%)
SVE1	8.96E+07	8.60E+07	95.98	8.42E+07	93.89
SVE2	8.31E+07	7.98E+07	96.1	7.78E+07	93.66
SVE3	1.28E+08	1.24E+08	97.1	1.22E+08	95.55
SVM1	8.17E+07	7.84E+07	95.94	7.59E+07	92.89
SVM2	8.55E+07	8.21E+07	96.05	7.99E+07	93.49
SVM3	1.39E+08	1.35E+08	97.1	1.33E+08	95.43
UGE1	1.02E+08	9.77E+07	95.64	9.57E+07	93.6
UGE2	5.03E+07	4.88E+07	97.09	4.72E+07	93.91
UGE3	1.24E+08	1.20E+08	97.06	1.18E+08	95.57
UGM1	1.31E+08	1.27E+08	97.13	1.25E+08	95.44
UGM2	7.98E+07	7.66E+07	96.03	7.40E+07	92.83
UGM3	8.57E+07	8.22E+07	95.94	8.00E+07	93.35

Table S2. Mapped read count matrix for each sample RNA-sequenced

Click here to download Table S2

Table S3. Differentially expressed genes in UGM relative to UGE (FDR≤0.05).

Click here to download Table S3

Table S4. Differentially expressed genes in SVM relative to UGE (FDR≤0.05).

Click here to download Table S4

Table S5. Differentially expressed genes in UGE relative to SVE (FDR≤0.05).

Click here to download Table S5

Development • Supplementary information

Table S6. Cell signalling genes upregulated in the UGM relative to the UGE.

Click here to download Table S6

Table S7. Cell signalling genes upregulated in the SVM relative to the UGE.

Click here to download Table S7

Table S8. Cell signalling genes upregulated in the UGE relative to the SVE.

Click here to download Table S8

Table S9. Cell signalling genes enriched in both UGM and SVM

Click here to download Table S9

Table S10. IPA predicted activated upstream genes enriched in UGM relative to UGE

Click here to download Table S10

Table S11. IPA predicted activated upstream genes enriched in UGE relative to SVE

Upstream Regulator	Molecule Type	Activation z-score	p-value of overlap
NR3C1	ligand-dependent nuclear receptor	2.56	1.86E-26
PTGER4	G-protein coupled receptor	4.22	1.58E-21
KLF2	transcription regulator	2.67	1.31E-20
IL10RA	transmembrane receptor	5.56	1.76E-20
SOX2	transcription regulator	2.60	9.54E-18
BCL6	transcription regulator	3.95	1.23E-14
SMAD7	transcription regulator	3.48	1.28E-14
SIRT1	transcription regulator	2.32	2.60E-14
PPARGC1A	transcription regulator	2.60	1.33E-11
BCL3	transcription regulator	3.25	3.90E-11
TRIM24	transcription regulator	3.59	8.12E-11
MEOX2	transcription regulator	3.53	1.18E-10
NOSTRIN	transcription regulator	4.19	9.78E-10
SIGIRR	transmembrane receptor	2.28	1.21E-09
NEUROG1	transcription regulator	2.75	3.11E-09
NKX2-3	transcription regulator	2.91	1.51E-08
GFI1	transcription regulator	3.17	2.07E-08
THRB	ligand-dependent nuclear receptor	3.19	3.64E-08
ZFP36	transcription regulator	3.40	5.24E-08
RARA	ligand-dependent nuclear receptor	2.84	5.43E-08
ACKR2	G-protein coupled receptor	3.44	6.04E-08
SP110	transcription regulator	2.72	9.35E-08
DACH1	transcription regulator	2.76	1.52E-07
ZNF281	transcription regulator	3.36	2.01E-07
GPS2	transcription regulator	3.21	1.12E-06
FEM1A	transcription regulator	2.61	1.80E-06
MYF5	transcription regulator	2.29	3.78E-06
TCF3	transcription regulator	2.55	1.83E-05
PRDM16	transcription regulator	2.33	2.01E-05
IKZF2	transcription regulator	2.67	2.89E-05
ADGRF5	G-protein coupled receptor	2.43	5.00E-05
IL1R2	transmembrane receptor	2.56	5.42E-05
CITED2	transcription regulator	2.99	6.27E-05
NR4A1	ligand-dependent nuclear receptor	2.29	6.37E-05
PIAS4	transcription regulator	2.65	8.70E-05
UNC5B	transmembrane receptor	2.43	1.35E-04
CBL	transcription regulator	3.06	3.74E-04
HNF1A	transcription regulator	2.76	6.47E-04
CIC	transcription regulator	2.43	1.10E-03
MAS1	G-protein coupled receptor	2.42	1.10E-03
GRHL3	transcription regulator	2.43	1.84E-03
ERF	transcription regulator	2.57	3.62E-03
HSR	G-protein coupled receptor	2.24	7.64E-03
Cux1	transcription regulator	2.24	1.60E-02
MAX	transcription regulator	2.97	1.33E-01
MYCN	transcription regulator	2.80	1.41E-01
ESRRA	transcription regulator	2.47	1.54E-01
HNF4A	transcription regulator	2.29	1.00E+00

Development • Supplementary information

Table S12. Ligand-receptor analysis

Click here to download Table S12

Table S13. Primers used for RT-qPCR

Target gene	Primer sequence
PSA (Forward)	CCTCACAGCTGCCCACTGCA
PSA (Reverse)	GATGAAACAGGCTGTGCCG
HPRT1 (Forward)	TGAACGTCTTGCTCGAGATGTG
HPRT1 (Reverse)	CCAGCAGGTCAGCAAAGAATTT

Development • Supplementary information
